# Strong correlation between protein reagent strip and protein-to-creatinine ratio for detection of renal dysfunction in HIV-infected patients: a cross-sectional study

**DOI:** 10.1186/s12981-015-0047-x

**Published:** 2015-03-21

**Authors:** José Ignacio López De León, Jose Antonio Mata-Marín, Karen Andrade-Fuentes, Gloria Huerta-Garcia, Juan C Domínguez-Hemosillo, Jesus Gaytán-Martínez

**Affiliations:** Nephrology Department, National Medical Center “La Raza”, IMSS, Mexico City, Mexico; Infectious diseases department, National Medical Center “La Raza”, IMSS, Mexico City, Mexico; Nephrology Department, HGR 25, IMSS, Mexico City, Mexico; Pediatric Hospital, National Medical Center “Siglo XXI”, IMSS, Mexico City, Mexico

**Keywords:** Protein reagent strip, Protein-to-creatinine ratio, Human immunodeficiency virus

## Abstract

**Background:**

Tubular dysfunction is common in HIV-infected people and detection of proteinuria is essential to identify this problem. In low-income countries, resources for detection of proteinuria using the Kidney Disease Improve Global Outcomes (KDIGO) gold standard urinary protein/creatinine ratio (uPCR) is rarely possible, and use of the protein reagent strip (PRS) could be an option in these places. The aims of this study were to establish the concordance between PRS and uPCR to detect tubular proteinuria in HIV-infected people, and to assess the sensitivity and specificity of PRS as a diagnostic method in this group.

**Methods:**

A cross-sectional study was conducted to evaluate the correlation between the two techniques to detect protein in urine. Participants were enrolled for a period of 6 months. The measurements were performed in participants who were on highly active antiretroviral therapy (HAART) or prior to the start of treatment. Proteinuria was defined as uPCR ≥ 150 mg/g, and/or ≥ trace on PRS. A phi coefficient was calculated to establish the degree of correlation. We assessed the sensitivity and specificity of PRS compared with uPCR using standard methods.

**Results:**

A total of 799 subjects were included. Of these, 737 (92%) were men. The mean age was 32.9 years (±10.1 years). Most (561, 70%) were on antiretroviral treatment. The mean estimated glomerular filtration rate (eGFR) calculated according to Modification of Diet in Renal Disease (MDRD)-4 was 113.0 mL/min (±22.6). Comorbidities included diabetes mellitus (10, 1.3%) and hypertension (17, 2.1%). The prevalence of proteinuria detected by PRS was 8.3% (n = 66) and by uPCR 10.6% (n = 85). The concordance assessed by phi correlation coefficient was 0.70, p < 0.001, with a sensitivity of 51.7% (95% confidence interval [CI] 41%–62%) and specificity 97% (95% CI 39%–97%).

**Conclusions:**

There is a high concordance between detection of proteinuria by PRS and uPCR. Therefore, in low-income countries PRS can be helpful for detecting tubular damage in people infected with HIV.

## Background

Kidney disease is a complication that is observed in 10%–30% of patients infected with human immunodeficiency virus (HIV), and is a common cause of morbidity and mortality [1,2]. In Mexico, an estimated 175,093 HIV-infected individuals are included in national registries [3]. The increased survival of HIV-infected patients has changed the course of the disease, with renal complications related to HIV now the fourth most common cause of mortality [4].

The prevalence of chronic kidney disease (CKD) in HIV-infected patients is 2%–10% [5], although some studies have estimated that 24% of HIV-infected patients have a baseline GFR < 90 mL/min [6,7]. Persistent proteinuria is the principal marker of glomerular and tubular disease in people with HIV/acquired immune deficiency syndrome (AIDS) and should be used for early identification of renal disease [8-10]. The Infectious Disease Society of America recommends that at the time of HIV diagnosis, all individuals should be assessed for kidney disease by a screening urine analysis for proteinuria, and that those with proteinuria should be referred for early treatment to prevent kidney failure [11].

To evaluate primary tubular and glomerular abnormalities, a specific and early screening process is required. It is generally accepted that measurement of the urine protein/creatinine ratio (uPCR) and the urine albumin/creatinine ratio (uACR) constitute a relatively inexpensive and effective method [12]. Proteinuria is defined as uPCR ≥ 150 mg/g on more than two occasions separated by 2–3 weeks [13]. The uPCR ratio was found to be 74.4%–90% sensitive and 93%–98% specific for the diagnosis of proteinuria of 0.5–2 g/day [14]. The protein reagent strip (PRS) can also be used to screen for proteinuria, and its sensitivity and specificity range from 80%–97% and 33%–80%, respectively [15]. In a rapid urine test, uPCR is preferred to uACR because it detects total urinary protein secondary to glomerular and tubular disease.

Some studies suggest that the use of test strips as a screening method increases by 13%–44% the likelihood of identifying a rapid deterioration in renal function in high-risk populations [16,17], dipstick false positive results have been found in association with dehydration (that increase the urine protein concentration, mainly albumin), exercise, hematuria, urinary tract infections, highly alkaline urines (pH 8); false-negative results are associated with excessive diuresis by overhydration and with proteins that do not have reactions with the dipstick (e.g. monoclonal proteins) [18]. The aims of this study were to establish a concordance between PRS and uPCR for detection of tubular proteinuria in HIV-infected people and to assess the sensitivity and specificity of PRS as a diagnostic method in this group.

## Results

A total of 799 HIV-infected patients were included, of whom 737 were men (92%). The mean age was 32.9 years (SD ± 10.1 years). Most participants (561, 70%) were on antiretroviral treatment.

The median eGFR calculated according to MDRD-4 was 113.06 mL/min (±22.62 mL/min), with 691 (86.48%) participants in MDRD-4 stage 1, 104 (13.01%) in stage 2, and 4 (0.50%) in stage 3. Ten participants (1.3%) had diabetes mellitus and 17 (2.1%) had hypertension (Table 1). The prevalence of proteinuria detected by dipstick was 8.3% (n = 66) and by uPCR was 10.6% (n = 85) [Table 2]. The concordance between PRS and uPCR assessed by the phi correlation coefficient was 0.7, p < 0.001.

**Table 1:** **Demographic characteristics of the study population**

**Characteristic total participants**	**Value n = 799 (100%)**
Sex (%)	
Male	737 (92.2%)
Female	62 (7.8%)
Age, years (mean ± SD)	32.9 ± 10.1
BMI (mean ± SD)	23.8 ± 2.8
Participants on HAART n (%)	561 (70.2%)
eGFR calculated using MDRD-4 mL/min (mean ± SD)	113.06 ± 22.6
Diabetes n (%)	10 (1.3%)
Hypertension n (%)	17 (2.1%)

BMI: body mass index; HAART: highly-active antiretroviral therapy; eGFR: estimated glomerular filtration rate; MDRD: modification of diet in renal disease.

**Table 2:** **Results**

	**uPCR significant**	**uPCR not significant**	**Total**
Proteinuria in PRS	44	22	66
No proteinuria in PRS	41	692	733
Total	85	714	799

When we evaluated the use of PRS to diagnose proteinuria, we found a sensitivity of 51.7% (95% confidence interval [CI] 41.3%–62%), specificity 97.2% (95% CI 95.3%–97.9%), positive predictive value 66.6% (95% CI 54.6%–76.8%), negative predictive value 94.4% (95% CI 92.5%–95.8%), positive likelihood ratio 18.4, and negative likelihood ratio 0.49 (0.52–0.94).

## Discussion

CKD is currently the fourth leading cause of morbidity and mortality in HIV-infected patients. One of the main factors determining progression of CKD is proteinuria from any source; therefore, it is important to apply diagnostic methods such as uPCR that allow us to identify proteinuria in HIV-infected people. Although this point is noted in the most recent European AIDS Clinical Society 2013 guidelines, unfortunately it is not always possible to carry out this testing in developing countries, where access to healthcare is limited. Not because of the reagent cost, but the uPCR test is not available in most of the clinical setting. For this reason it is important to evaluate other diagnostic options that are available to a greater number of people and medical centers, including diagnostic methods with high sensitivity and specificity that are comparable to the gold standard defined according to the Kidney Disease Improving Global Outcomes 2012 guidelines, such as the use of PRS for detecting proteinuria.

In our study, the population was composed mainly of men in the third decade of life, most of them with normal renal function at baseline, with proteinuria documented in approximately 10% by uPCR. We found a strong positive correlation between PRS and uPCR results and a high specificity and negative predictive value of PRS for detection of proteinuria in HIV-infected patients. These data suggest that a negative PRS result is useful to exclude disease and a positive PRS is associated with high probability of the presence of disease.

This result is consistent with other reports that PRS results are useful mainly when proteinuria exceeds 300–500 mg/day, that they correlate well with the uPCR, and have a low positive predictive value and a high negative predictive value [19-21]. However, Realston *et al*. reported that PRS testing had a sensitivity of 100% and a high incidence of false positives (48%) at low levels of proteinuria [22]. The high sensitivity in that study was linked to the methodology used, as only patients with a trace or more of protein were included, in contrast to our study in which all participants with or without traces of protein were included, allowing a better determination of the correlation between the two diagnostic methods.

In another study in pregnant women, the positive predictive value of PRS ranged from 64.9% (single voided urine sample) to 94.2% (24-hour urine aliquot) and the negative predictive value ranged from 75.2% (single voided urine sample) to 84.2% (24-hour urine aliquot), with the authors concluding that PRS had low accuracy as a screening method [23].

The usefulness of proteinuria determined by PRS as a risk factor for development and progression of chronic kidney disease is supported by the data published by Iseki *et al.* [24] and Gomez de Mattos Cavalcante *et al.* [25], who demonstrated that proteinuria assessed by PRS is an independent risk factor for end-stage renal disease, with a cumulative incidence of 1.4% with proteinuria (1+) and 7.1% with proteinuria (2+) at 17 years of follow-up.

The main strength of this study is the population size and the homogeneity of the sample: most of the population constituted men in the third decade of life with normal renal function, only four had stage 3 CKD, and there was a low prevalence of comorbidities at the time of the study. Statistical analysis supports the conclusion that there is a high correlation between quantitative and qualitative methods for the detection of proteinuria, with a phi correlation coefficient close to one. We also evaluated the sensitivity and specificity of PRS in HIV-infected patients with robust statistical likelihood testing, which provides a qualitative determination of the high value of detecting the presence of protein in urine. Most of the previously mentioned studies included healthy or pregnant individuals or those with various comorbidities including nephropathy, autoimmune diseases, and others. Only a few studies have focused on the analysis of proteinuria and diagnostic methods and their effectiveness in HIV-infected patients [26,27], which made similar conclusions to ours, considering PRS a method with low sensitivity but with a good correlation with uPCR, due to the presence of different proteins, instead of albumin. Siedner *et al*. reported greater sensitivity with a higher uPCR (≥300 mg/g); however, in our study the cut-off point was 150 mg/g, therefore, the sensibility is compromised but the specificity is better.. However because of the similarity with previous studies we expect a similar behavior, so considering 300 mg/g as a cutoff we would expect that the positive predictive value and sensitivity also increase.

Guidelines for the management of chronic kidney disease in patients with HIV issued by the Infectious Diseases Society of America recommend that urine dipstick tests should be used to detect kidney abnormalities in this population [1].

This test should have the ability to detect kidney disease with a threshold of 1+ proteinuria, but dipstick tests measure levels of albumin, and if urine is diluted the accuracy of these tests is affected. Furthermore, some types of kidney disease, including tubular disorders, cannot be accurately diagnosed using dipstick tests.

The majority of the participants in this study has a preserved GFR, indicating a minimal glomerular damage, so the proteinuria would mainly be tubular not glomerular.

We did not find any study in Mexico addressing PRS as a diagnostic test in HIV-infected individuals, a question that we believe is very important because of the high risk of proteinuria in this specific population.

Regarding the limitations of the study, first, measurement of proteinuria by PRS is susceptible to errors depending on the observer, sample handling, and the quality of the test strips. Additional participant information including the type of antiretroviral treatment and its duration, or the time from diagnosis of the disease, were not considered in this study, which focused only on the correlation between two diagnostic methods and not on factors associated with the onset of proteinuria. Further studies evaluating these points should be considered. We could not evaluate the type of antiretroviral therapy with the PRS or uPCR, in addition, the association of TDF with the abnormalities found in this test was not measured.

Finally, based on the results of this study, we suggest that the correlation between uPCR and PRS is strong enough to consider the latter a cost-effective diagnostic method applicable to our population.

In future studies is necessary to take in to consideration the degree of proteinuria in dipstick, classifying according to the number of + of proteins, as well as increasing uPCR cut off point and avoiding or decreasing the possibility of false negative or positive results knowing the clinical state of the patients at the moment of sampling.

## Conclusions

There is a high concordance between detection of proteinuria by PRS and uPCR with a fair sensitivity, high specificity, and negative predictive values for PRS. Although the qualitative method is not better for screening than the quantitative method, in low-income countries, PRS analysis can be a useful tool to detect tubular damage in HIV-infected individuals.

## Methods

### Study design

After obtaining approval for the research protocol from the Ethics and Investigation Committee of “La Raza” National Medical Center (approval number R-2014-3502-147), we conducted a cross-sectional study between August 2012 and December 2013 at Hospital de Infectología “La Raza” in Mexico City.

### Participants

The sample included treatment-naïve and treatment-experienced HIV-infected patients aged between 17 and 79 years who attended the HIV clinic and who underwent PRS and quantitative urine protein determinations, treatment regimen was not recorded. We excluded individuals with overt hematuria. Individuals were also excluded if they withdrew their informed consent. After obtaining informed consent, each participant was interviewed, and information including age and current chronic diseases was recorded on the designated record forms.

### Measurements

The enrolled participants were assessed for weight, height, and antiretroviral treatment. Serum creatinine was measured by the chemiluminescence method. Urine creatinine was measured quantitatively using a photometric method, and urine total protein was measured quantitatively using a turbidimetric procedure; a Multistix 10 SG (SIEMENS®) reagent strip was used for qualitative assessment, evaluating the presence of proteinuria, with 1+ or more. Estimated glomerular filtration rate (eGFR) in mL/min was calculated using the Modification of Diet and in Renal Disease-4 (MDRD-4). We defined proteinuria as uPCR ≥ 150 mg/g and ≥ trace on PRS, the time between samples for each test was less than 24 h, and in most of the patients both tests were done with the same urine sample.

### Statistical analysis

Statistical analysis was performed using the Statistical Package for Social Sciences (SPSS) software, version 21 (IBM, Armonk, NY). Descriptive statistics were used to evaluate the demographic characteristics of the study population; because the continuous variables that were analyzed were normally distributed, the mean and standard deviation were used. A phi coefficient was calculated to establish the degree of correlation. Using standard methods we assessed the sensitivity, specificity, and positive and negative predictive values of PRS by comparison with uPCR as a gold standard. A value of p < 0.05 was considered significant.
